# The minimum mass ratio of W UMa-type contact binaries—a new calculation

**DOI:** 10.1038/s41598-024-63833-y

**Published:** 2024-06-06

**Authors:** Xu-Dong Zhang

**Affiliations:** School of Physics and Electronic Science, Guizhou Normal University, Guiyang, 550025 China

**Keywords:** Eclipsing binaries, W UMa, Minimum mass ratio, Astronomy and astrophysics, Astronomy and astrophysics

## Abstract

A model is developed to establish the relationship between the critical gyration radius *k* of the primary component and the mass ratio (*q*) by considering the different dimensionless gyration radii of main-sequence stars with varying masses. The next step involves obtaining the low mass ratio limit (${q_{\text{min}}} = 0.038 \sim 0.041$ for overcontact degree $f = 0$~ 1) of W UMa-type contact binaries. Furthermore, the radial density distributions are estimated within the range of $0.3 M_{\odot } \sim 4.0 M_{\odot }$, based on the mass-radius relationship of main-sequence stars. Subsequently, the physical meaning of the minimum *k* value is proposed, which leads to an explanation for the cause of the minimum mass ratio. Finally, a stability criterion is proposed, which is based on both the mass ratio (*q*) and the total mass of the two components ($M_{tot}$).

## Introduction

Since Hut (1980)^1^ demonstrated that a binary system can only be in tidal equilibrium if the orbital angular momentum exceeds three quarters of the total angular momentum, researchers have attempted to estimate the minimum mass ratio of contact binaries by analyzing the dynamical stability of a binary system. However, accurately calculating the dimensionless gyration radius *k* of the components poses significant challenges. Previous studies have relied on approximations to determine the minimum mass ratio. Rasio (1995)^2^ predicted a cut-off mass ratio for W UMa systems at approximately 0.09 by neglecting the angular momenta of secondary components. Li and Zhang (2006)^3^ obtained a minimum mass ratio for stable contact binaries at around 0.071, by considering rotation in secondaries and assuming $k_2^2=k_1^2=0.06$. Arbutina (2007, 2009)^4,5^ introduced a new criterion ($\frac{\textrm{dJ}_{\textrm{tot}}}{\textrm{da}}$=0) and assumed $k_2^2 \ne k_1^2$ for stability by accounting for distortion caused by rotation and companionship, resulting in lower values for the minimum mass ratio at 0.070. Landin et al. (2009)^6^ modelled gyration radii values for low mass pre-main sequence stars, providing more robust and comprehensive criteria for the critical instability mass ratio of contact binaries. It should be emphasized that only primary components enable estimation of gyration radii values with masses $M_{1}$. This is because the primary components of contact binaries are close to the main sequence stars in terms of Mass-Radius and Mass-Luminosity relations, in contrast, secondary components appear brighter and larger than main sequence stars with the same mass^7–9^. Additionally, a typical W UMa-type contact binary usually has short period and low mass^10,11^. Wadhwa et al. (2021)^12^ derived a criteria for the critical instability mass ratio of contact binaries with different masses of the primary components. However, no simple and effective criterion to judge the stability of contact binaries has been established, and the minimum mass ratio problem remains unsolved.

The mass-luminosity, mass-radius, and mass-effective temperature relations (MLR, MRR and MTR) of 509 main-sequence stars were studied by Eker et al. (2018)^13^. Their findings provide a foundation for further investigation into the physical nature of minimum mass ratio. In this paper, we firstly analyzed the relation between mass ratio *q* and the gyration radius of primary component $k_{1}$ for a stable contact binary system ($\frac{\textrm{dJ}_{\textrm{tot}}}{\textrm{da}}$=0). Then, we analyzed the radial density distributions of main sequence stars with different mass and found the mass break point of $1.5 M_{\odot }$ which in agreement with Landin et al. (2009)^6^. Finally, we established a new lower limit for mass ratio in contact binaries and discussed a stability criterion based on both the mass ratio *q* and the total mass of the two components $M_{tot}$.

## Calculation

From the condition $\frac{\textrm{dJ}_{\textrm{tot}}}{\textrm{da}}=0$ (where $J_{\text{tot}} = J_{\text{orb}} + J_{\text{spin}}$)^4^, the critical mass ratio for a fixed fill-out factor ($0\le f \le 1$) can be calculated numerically. According to the Kepler’s third law, the orbital angular momentum ($J_{\text{orb}}$) of a binary system can be expressed as

1 $$\begin{aligned} {J_{\text{orb}}}=\frac{M_{1}M_{2}}{M_{1}+M_{2}}\omega a^{2}=q\sqrt{\frac{GM_{1}^{3}a}{1+q}}=\frac{q\sqrt{G(M_{1}+M_{2})^{3}a}}{(1+q)^{2}}, \end{aligned}$$

where $M_{1}$ and $M_{2}$ are the masses of the primary and secondary components, *q* is the mass ratio ($M_{2}/M_{1}$), *a* is the separation between two components, and $\omega$ is the orbital angular velocity. The spin angular momentum ($J_\text{spin}$) of a binary is described by

2 $$\begin{aligned} {J_\text{spin}} = k_{1}^{2}M_{1}R_{1}^{2}\omega + k_{2}^{2}M_{2}R_{2}^{2}\omega =\sqrt{G(M_{1}+M_{2})^{3}a}\bigg[k_{1}^{2}\bigg(\frac{1}{1+q}\bigg)\bigg(\frac{R_{1}}{a}\bigg)^{2} + k_{2}^{2}\bigg(\frac{q}{1+q}\bigg)\bigg(\frac{R_{2}}{a}\bigg)^{2}\bigg], \end{aligned}$$

where $k_{1}$ and $k_{2}$ are the gyration radii of the two components, $R_{1}$ and $R_{2}$ are radii of two components. In a contact binary system, the stellar radii of the two components ($R_{1}$, $R_{2}$) are roughly the size of the equipotential surface volume radii passing through the Lagrangian points L1 (inner Roche lobe, $R_\text{IL}$) and L2 (outer Roche lobe, $R_\text{OL}$). The effective radii of the inner and outer Roche lobes are typically determined using the approximations of Eggleton (1983)^14^ and Yakut and Eggleton (2005)^7^, respectively. Consequently, the effective radii of primaries and secondaries can be estimated using the following equations,

3 $$\begin{aligned} \frac{R_\text{ILi}}{a}= \left\{ \begin{array}{lr} \frac{0.49q^{-2/3}}{0.6q^{-2/3}+\textrm{ln}(1+q^{-1/3})} &{} \mathrm i=1 \\ \frac{0.49q^{2/3}}{0.6q^{2/3}+\textrm{ln}(1+q^{1/3})} &{} \mathrm i=2 \end{array} \right. \end{aligned}$$

and

4 $$\begin{aligned} \frac{R_\text{OLi}}{a}= \left\{ \begin{array}{lr} \frac{0.49q^{-2/3}+0.15}{0.6q^{-2/3}+\textrm{ln}(1+q^{-1/3})} &{} \mathrm i=1 \\ \frac{0.49q^{2/3}+0.27q-0.12q^{4/3}}{0.6q^{2/3}+\textrm{ln}(1+q^{1/3})} &{} \mathrm i=2 \end{array}{.} \right. \end{aligned}$$

The fill-out factor (*f*) is defined as,

5 $$\begin{aligned} f=\frac{\Omega - \Omega _\text{IL}}{\Omega _\text{OL} - \Omega _\text{IL}} \approx \frac{R - R_\text{IL}}{R_\text{OL} - R_\text{IL}}{,} \end{aligned}$$

where $\Omega$ is the surface potential of the star, $\Omega _\text{IL}$ and $\Omega _\text{OL}$ are the surface potential of the inner and outer lobes, respectively. Similarly, *R* is the radius of the star, $R_\text{IL}$ and $R_\text{OL}$ are the radii of the inner and outer lobes, respectively. By combining Eqs. (3)–(5), Arbutina (2007)^4^ obtained:

6 $$\begin{aligned} R_{2} = P(q)a + Q(q)R_{1}, \end{aligned}$$

where

7 $$\begin{aligned} {\begin{matrix} Q(q)= \frac{R_\text{OL2}-R_\text{IL2}}{R_{\rm OL1}-R_{\rm IL1}}\\ P(q)= \frac{R_{\rm IL2}}{a}-Q(q)\frac{R_{\rm IL1}}{a}{.} \end{matrix}} \end{aligned}$$

Then the total angular momentum of a contact binary can be written as

8 $$\begin{aligned} J_{\rm tot} = \frac{q\sqrt{GM^{3}R_{1}}}{(1+q)^{2}}\sqrt{\frac{a}{R_{1}}} \Bigg \{ 1+\frac{k_{1}^{2}(1+q)}{q} \Bigg [ \left( 1+\frac{k_{2}^{2}}{k_{1}^{2}}qQ^{2}\right) \left( \frac{R_{1}}{a} \right) ^{2}+2\frac{k_{2}^{2}}{k_{1}^{2}}qPQ \left( \frac{R_{1}}{a} \right) +\frac{k_{2}^{2}}{k_{1}^{2}}qP^{2} \Bigg ] \Bigg \}, \end{aligned}$$

from the condition $\frac{\textrm{dJ}_{\textrm{tot}}}{\textrm{da}}=0$, the critical separation is given by

9 $$\begin{aligned} \frac{R_{1}}{a} = \frac{\frac{k_{2}^{2}}{k_{1}^{2}}qP^{2}+\frac{q}{k_{1}^{2}(1+q)}}{\frac{k_{2}^{2}}{k_{1}^{2}}qPQ+\sqrt{(\frac{k_{2}^{2}}{k_{1}^{2}}qPQ)^2+3(1+\frac{k_{2}^{2}}{k_{1}^{2}}qQ^{2})(\frac{k_{2}^{2}}{k_{1}^{2}}qP^{2}+\frac{q}{k_{1}^{2}(1+q)})}}. \end{aligned}$$

By combining Eqs. (3)–(5), one can find

10 $$\begin{aligned} \frac{R_{1}}{a} = \frac{(R_{\rm OL1}-R_{\rm IL1})\times f+R_{\rm IL1}}{a} = \frac{0.49q^{-2/3}+0.15f}{0.6q^{-2/3}+\mathrm ln(1+q^{-1/3})}. \end{aligned}$$

**Figure 1:**
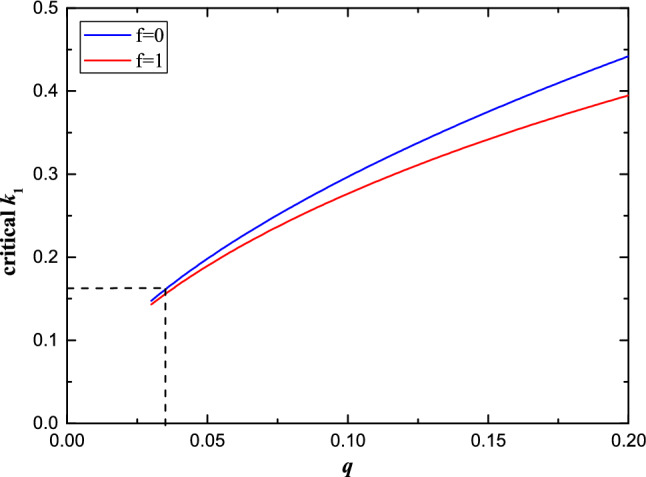
Relationship between critical $k_{1}$ and *q*. For a stable binary system with a specific mass ratio *q*, the gyration radius of primary component $k_{1}$ should less than a critical value.

The secondaries in extremely low mass ratio contact binaries are very low mass stars, indicating that $k_{2}^{2} \approx 0.205$ (n = 1.5 polytrope—fully convective stars). Based on the above derivation and assumptions (details can also be found in Arbutina 2007^4^ and Wadhwa et al. 2021^12^), the critical $k_{1}$ can be obtained for a fixed mass ratio *q* by using Eq. (9) and (10). The results of the calculation are depicted in Fig. 1.

**Figure 2:**
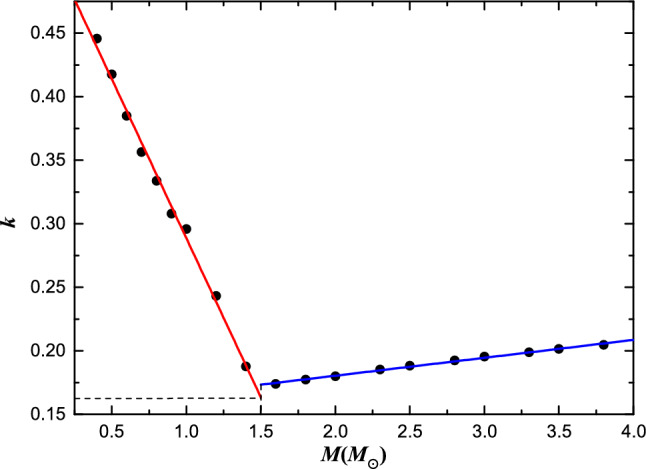
The values of gyration radii *k* calculated with different masses were modelled by Landin et al. (2009)^6^. The red and blue lines represent the linear fits of masses above and below 1.5 $M_{\odot }$, respectively.

The gyration radii *k*, including effects of rotation and tidal distortion due to binary interaction are determined by Landin et al. (2009)^6^. According to their data, two linear relationships for different mass ranges are shown in Fig. 2, and the fitting formulas are as follows,

11 $$\begin{aligned} \left\{ \begin{array}{lr} k = 0.5391-0.2504\times M &{} (0.4 M_{\odot } \leqslant M \leqslant 1.5 M_{\odot }), \\ k = 0.1522+0.0141\times M &{} (1.5 M_{\odot } < M \leqslant 4.0 M_{\odot }). \end{array} \right. \end{aligned}$$

The break point of the two fitted curves indicates that *k* has a minimum value, as seen in Fig. 2. Considering the relationship between *k* and *q* for a stable contact binary system (see Fig. 1), *q* also has a minimum value. After calculating the value $k \approx 0.164$ at a mass of 1.5 $M_{\odot }$, the minimum mass ratio can be easily obtained (${q_{\rm min}} = 0.038 \sim 0.041$ for overcontact degree $f = 0 \sim 1$).

## Discussions

### Potential reasons for the minimum *k* value

**Figure 3:**
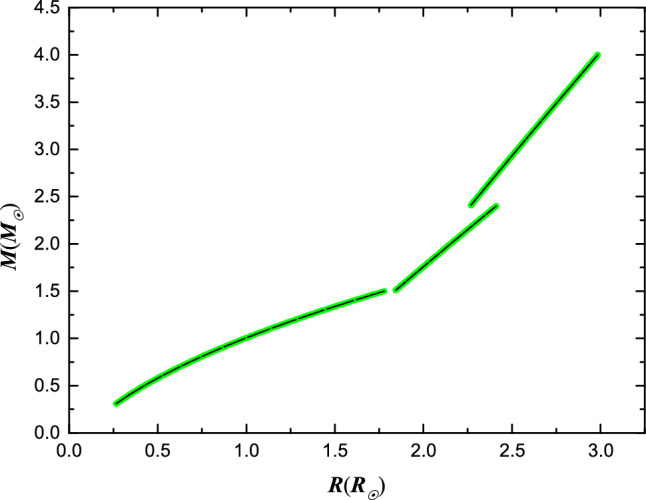
Recomputed mass-radius relation in the range of $0.3 M_{\odot }$ to $4.0 M_{\odot }$ by using the mass-luminosity and mass-effective temperature relations given by Eker et al. (2018)^13^. The black lines represent the fitted lines using Eq. (13) proposed by Zhang et al. (2020)^9^.

The results from Landin et al. (2009)^6^ reveals the existence of a minimum value for *k* within the star mass range of $1.4 M_{\odot }$ to $1.6 M_{\odot }$, as illustrated in Fig. 2. In order to delve into the physical implications of this minimum *k* value and explore phenomena occurring in the mass range $M = 1.4 \sim 1.6 M_{\odot }$, an analysis of the mass-radius relation and radial density distribution of main-sequence stars is conducted. This investigation aims to dissect the interior structure of main-sequence stars, shedding light on the underlying mechanics at play within this critical mass range. Eker et al. (2018)^13^ calibrated the mass-luminosity relation in the mass range $0.179 \sim 31 M_{\odot }$, mass-radius relation in the mass range $0.179 \sim 1.5 M_{\odot }$ and mass-effective temperature relation in the mass range $1.5 \sim 31 M_{\odot }$. The remaining part of mass-radius relation was completed using Stefan–Boltzmann law ($L = 4\pi R^{2}\sigma T^{4}$). Figure 3 illustrates the mass-radius relation within a mass range of $0.3 \sim 4.0 M_{\odot }$. Zhang et al. (2020)^9^ proposed an approximate formula to describe the radial variation of density,

12 $$\begin{aligned} \rho =\delta \cdot r^{\beta }, \end{aligned}$$

where *r* is the radial variation of radius, the unit of *r* adopts star radius *R*, $\delta$ and $\beta$ are dimensionless parameters, and the unit of $\rho$ then becomes $M_{\odot }/R_{\odot }^{3}$. Then the mass-radius relation based on radial density distribution can be written as follows,

13 $$\begin{aligned} M = \frac{4\pi }{3+\beta } \delta \cdot R^{3+\beta }. \end{aligned}$$

**Figure 4:**
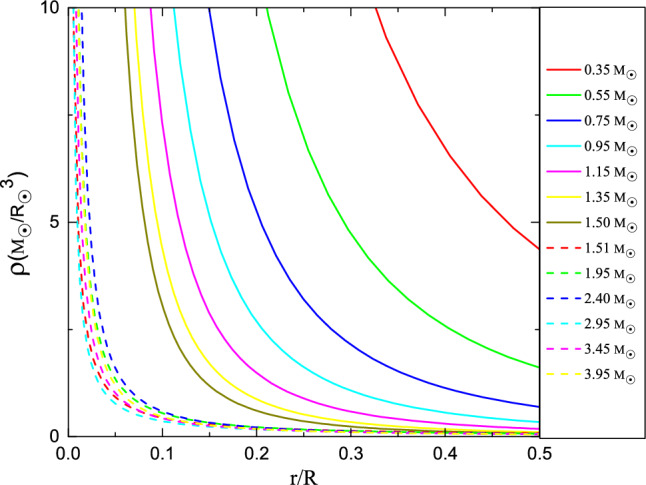
Thirteen examples of star density distributions are calculated using Eq. (12). A trend towards more concentrated density distributions is displayed when a star’s mass exceeds $1.5 M_{\odot }$.

Equation (13) is the accepted form of describing the mass-radius relationship and can be derived in other ways. Thus, based on the conjecture of the expression form of Eq. (13), Eq. (12) is proposed to explore the radial density distribution of the star. It is not difficult to find that the density at the center tends to infinity, however, as it move closer to the center, the volume proportion becomes smaller. Consequently, the mass proportion of this region can be ignored without affecting the mass-radius relationship. For instance, in a star with a mass of 1.4 $M_{\odot }$, the mass within a thousandth radius of the center accounts for less than 1% of the total mass, falling within the error margin of the star mass measurable by current technology. This value becomes even more negligible for stars with masses exceeding 1.5 $M_{\odot }$. Therefore, despite the current drawbacks of Eq. (12), we believe that this will have no impact on our subsequent discussion, as all our assumptions are based on the mass–radius relationship. Comparing Eq. (13) with the recomputed mass–radius relation, a series of $\delta$s and $\beta$s are obtained in the range of $0.3 M_{\odot }$ to $4.0 M_{\odot }$ (see Table 1), and the fitted lines are shown in Fig. 3. By utilizing $\delta$ and $\beta$ values, density distributions can be calculated for stars across different mass ranges. In Fig. 4, thirteen examples are presented to illustrate the phenomenon under discussion. When a star’s mass surpasses $1.5 M_{\odot }$, there is a discernible trend towards more concentrated density distributions, as depicted in Fig. 4. The transition of nuclear reactions from the p–p chain to the CNO cycle is characterized by a requirement of higher temperatures for the latter compared to the former. Therefore, the mass turning point when CNO cycle reactions gradually takeover p–p chain is expected at around $1.5 M_{\odot }$. Consequently, the distinct break point observed in Fig. 3 could potentially be ascribed to the varying radial density distributions (or the different structures) exhibited by stars with masses below and above $1.5 M_{\odot }$. This is consistent with the finding that stars’s radiative-dominated and convective-dominated envelopes diverged at around 1.5 $M_{\odot }$^15,16^.

**Table 1:** The values of $\delta$ and $\beta$ in Eqs. (12) and (13) at different mass range.

Mass range ($M_{\odot }$)	$\delta$	$\beta$	Mass range ($M_{\odot }$)	$\delta$	$\beta$
0.31–0.4	0.10965	$-1.92875$	2.21–2.3	0.07499	$-1.40924$
0.41–0.5	0.08809	$-2.03440$	2.31–2.4	0.07551	$-1.44102$
0.51–0.6	0.07587	$-2.11162$	2.41–2.5	0.07173	$-0.88462$
0.61–0.7	0.06907	$-2.16359$	2.51–2.6	0.07307	$-0.93610$
0.71–0.8	0.06465	$-2.20276$	2.61–2.7	0.07434	$-0.98329$
0.81–0.9	0.06159	$-2.23345$	2.71–2.8	0.07556	$-1.02674$
0.91–1.0	0.05939	$-2.25819$	2.81–2.9	0.07672	$-1.06691$
1.01–1.1	0.05776	$-2.27860$	2.91–3.0	0.07784	$-1.10417$
1.11–1.2	0.05651	$-2.29575$	3.01–3.1	0.07892	$-1.13885$
1.21–1.3	0.05554	$-2.31037$	3.11–3.2	0.07996	$-1.17123$
1.31–1.4	0.05478	$-2.32299$	3.21–3.3	0.08097	$-1.20154$
1.41–1.5	0.05417	$-2.33401$	3.31–3.4	0.08195	$-1.22998$
1.51–1.6	0.07121	$-1.07269$	3.41–3.5	0.08289	$-1.25675$
1.61–1.7	0.07176	$-1.13872$	3.51–3.6	0.08381	$-1.28198$
1.71–1.8	0.07230	$-1.19687$	3.61–3.7	0.08471	$-1.30583$
1.81–1.9	0.07284	$-1.24856$	3.71–3.8	0.08558	$-1.32841$
1.91–2.0	0.07339	$-1.29487$	3.81–3.9	0.08643	$-1.34982$
2.01–2.1	0.07392	$-1.33666$	3.91–4.0	0.08726	$-1.37016$
2.11–2.2	0.07446	$-1.37460$			

### Study on the stability of the extremely low mass ratio contact binaries from previous papers

**Figure 5:**
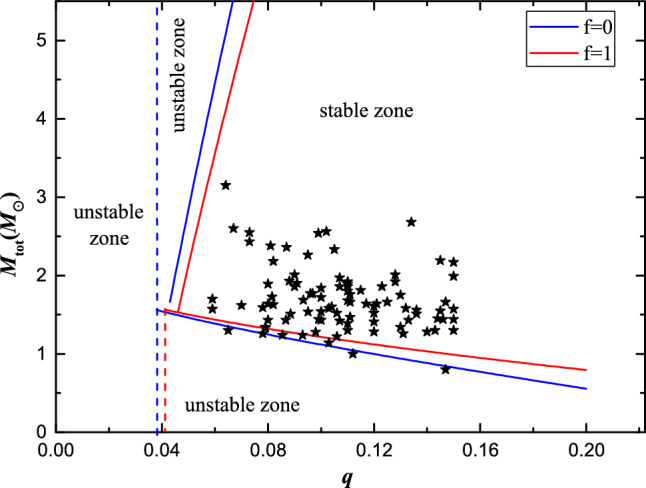
$q-M_{\text{tot}}$ map for low mass ratio W UMa-type contact binaries. The blue and red lines represent critical masses with respect to mass ratios for stable systems. The dashed lines refer to minimum mass ratio. Contact binary systems can be stable only if total masses are in certain ranges with respect to different mass ratios.

Furthermore, based on our analysis mentioned earlier, an extremely low mass ratio contact binary can only remain stable if *q* and $k_{1}$ are combined in specific ways. Specifically, for a given *q* value, $k_{1}$ must be less than the critical value determined by using Eqs (9) and (10). Consequently, it becomes relatively straightforward to obtain critical masses (blue and red lines in Fig. 5) using Eq. (11). Finally, four zones are divided according to stability conditions mentioned above (see Fig. 5):

*a*, unstable zone, where no stable contact binary systems exist when the mass ratio falls below the minimum value (0.038);

*b*, unstable zone for total masses less than $1.56 M_{\odot }$, when $M_{\rm total}$ lie below the critical lines, $k_{1}$ and *q* cannot satisfy $\frac{\textrm{dJ}_{\textrm{tot}}}{\textrm{da}}$=0;

*c*, unstable zone for total masses greater than $1.56 M_{\odot }$, similar to *b* zone but $M_{\rm total}$ lie above the critical lines;

*d*, stable zone, where low mass ratio W UMa-type contact binaries reside on this region of $q - M_{\rm tot}$ map satisfying current stability condition.

Recently compiled data from Latkovic et al. (2021)^11^, Christopoulou et al. (2022)^17^ and Liu et al. (2023)^18^ includes numerous samples featuring a mass ratio lower than 0.15 which were selected specifically for analyzing their current stability status. The results indicate that all these extremely low mass ratio contact binaries are located in the stable zone(see Fig. 5). In summary, all the extremely low mass ratio contact binaries observed so far satisfy the dynamical stability condition until their mass ratios decrease to critical values due to angular momentum loss or mass transfer.

## Conclusions and outlook

Combining the result of Arbutina (2007)^4^, we have derived the relationship between critical gyration radius of primary component $k_{1}$ and mass ratio *q* for contact binaries. The extremely low mass ratio contact binaries are stable only if gyration radii of primary components $k_{1}$ less than critical values. It should be noted that there exists a minimum value of *k* within the range of $1.4 \sim 1.6 M_{\odot }$. This minimum *k* value is possibly caused by the mass break point ($1.5 M_{\odot }$) in radial density distributions of main sequence stars with different masses, suggesting that CNO cycle reactions gradually takeover p-p chain for stars more massive than $1.5 M_{\odot }$. Then, a minimum critical mass ratio (${q_{\rm min}} \approx 0.038$) is obtained based on the above analyses. Additionally, a new criterion based on both the mass ratio *q* and the total mass of the two components $M_{tot}$ has been proposed. Contact binaries with extremely low mass ratios can be stable as long as their total masses of two components are within a certain range. Therefore, it should be noted that ${q_{\rm min}}$ is a global minimum, and the instability mass ratio is dependant on the total mass for each unique system^19^. Finally, we investigated recent extremely low mass ratio contact binaries collected by previous researchers and found that they all meet the stability condition so far.

When contact binaries are at the boundary of Fig. 5, mass transfer and mass loss will determine whether these binary systems continue to evolve to merge. Therefore, it is very important to study the mass transfer and mass loss of the contact binaries on the boundary. For a given binary system with known initial mass, initial mass ratio, and initial orbital period, combined with the critical mass ratios, Ge et al.^15,16,20,21^ obtained the possible evolutionary channel, either dynamically stable/unstable mass transfer or thermally stable/unstable mass transfer or thermal equilibrium mass loss, this series of studies will be of guiding significance for the subsequent research.

## Data Availability

Some datasets analyzed in this study can be accessed at https://wumacat.aob.rs, please cite^11^ when using them. Others are available from the corresponding author on reasonable request.
